# A One-Year Cross-Sectional Study on the Impact of the Thyroid Imaging Reporting and Data System (TIRADS) on Fine-Needle Aspiration Cytology (FNAC) Decision-Making in a Secondary Care Hospital

**DOI:** 10.7759/cureus.89623

**Published:** 2025-08-08

**Authors:** Srinivasa Swamy Bandaru, Qahtan A Al Dulaimi

**Affiliations:** 1 General Surgery, Saqr Hospital, Emirates Health Services, Ras Al Khaimah, ARE; 2 Surgery, RAK Medical and Health Sciences University, Ras Al Khaimah, ARE

**Keywords:** bethesda scoring, fine-needle aspiration cytology, histopathology, malignancy, surgery, thyroid imaging reporting and data system

## Abstract

Introduction: The widespread utilization of neck ultrasound (US) by family physicians for the investigation of non-specific neck symptoms, as well as by endocrinologists and general surgeons for symptomatic thyroid problems, has led to an increase in the detection of nonpalpable thyroid nodules. This presents challenges and dilemmas regarding the decision to perform fine-needle aspiration cytology (FNAC). The routine use of cytology is often considered unnecessary, costly, and inconvenient for patients. Therefore, this observational study was conducted to explore how FNAC could be avoided by using the Thyroid Imaging Reporting and Data System (TIRADS), drawing insights from comparative data analysis with the Bethesda cytology system and postoperative histopathology.

Objective: This study aims to observe trends in decision-making for performing FNAC using only the US TIRADS scoring, without considering nodule size, by comparing the results with histopathology of the operated patients and recommending conclusions.

Material and methods: Data were collected over a one-year period from January 1, 2023, to December 31, 2023, from the hospital's electronic medical records, resulting in 89 cases for analysis and comparison. All thyroid nodule cases with a US TIRADS score were included in the study, while post-total thyroidectomy cases as follow-up and patients who died during the study period were excluded.

Results: Among the 89 cases, US-guided FNAC was performed in 37 cases (41.5%). In 38 cases (42.5%), FNAC was not recommended by the clinician, while in the remaining cases, it was advised but not performed due to patient refusal (27.5%). For TIRADS score 1, no FNAC was performed. In TIRADS score 2, FNAC was conducted in two out of 19 cases, both confirmed as benign. For TIRADS score 3, among 22 FNAC cases, one was categorized as Bethesda 3, while the rest had Bethesda scores of 2 or lower; none underwent surgery. In TIRADS score 4, FNAC was performed in 12 cases, with four classified as Bethesda 4, all of whom underwent surgery. One of these cases was confirmed as malignant. In TIRADS score 5, a single case was identified, which yielded a Bethesda 5 result on FNAC and was later confirmed as malignant on postoperative histopathology. Overall, 37 out of 89 cases (41.5%) underwent FNAC, with five cases proceeding to surgery (13%). Histopathological analysis confirmed malignancy in two cases, resulting in an overall malignancy rate of 5.5% among FNAC cases.

Conclusions: Patient reluctance toward FNAC due to concerns over discomfort and complications remains a barrier. FNAC is generally not indicated for TIRADS categories 1 and 2. In our study, FNAC appeared unwarranted for TIRADS category 3, as none of these patients underwent surgery despite constituting 60% of the cohort, aligning with literature that reports a malignancy risk of under 5% in this category. Such nodules may be better managed through periodic surveillance and individualized risk stratification based on factors such as patient age and nodule size. In contrast, FNAC was justified and appropriately applied in TIRADS 4 and 5 categories, which carry a higher risk of malignancy. Although nodule size was not analyzed in this study, the results are consistent with previously established data.

## Introduction

The study utilized the comparative data outcomes of the ultrasound (US) Thyroid Imaging Reporting and Data System (TIRADS) [1], the Bethesda cytology grading method [2], and postoperative histopathology in predicting malignancy risks in thyroid nodules based on TIRADS score alone, so as to avoid unwarranted fine-needle aspiration cytology (FNAC).

This observational study was conducted in accordance with the Strengthening the Reporting of Observational Studies in Epidemiology (STROBE) guidelines [3], which provide a structured framework for transparently reporting the design, execution, and outcomes of observational research. The study focuses on the increasing detection of non-palpable thyroid nodules within the general population served by a secondary care hospital in the United Arab Emirates (UAE). This trend is largely driven by the widespread use of neck US by family physicians for nonspecific neck concerns, as well as routine thyroid US screenings by endocrinologists for cases of thyroid dysfunction. These findings are in addition to patients presenting with clinically apparent thyroid enlargement [4,5].

While only 5-10% of thyroid nodules are typically malignant, the diagnostic process often relies on FNAC to guide surgical decision-making [5-7]. However, determining which nodules warrant FNAC remains a significant challenge for surgeons in outpatient settings. This study aims to evaluate one year of cross-sectional data from patients with thyroid nodules at a secondary care hospital in the UAE. Specifically, it seeks to compare TIRADS with FNAC decision-making and assess their correlation with postoperative histopathology findings, taking into consideration available recommendations in the literature [5-7]. Descriptive statistics were used to report frequencies and percentages. Sensitivity, specificity, positive predictive value (PPV), and negative predictive value (NPV) were calculated for TIRADS 3, 4, and 5 against histopathology.

## Materials and methods

This retrospective study utilized patient data extracted from the hospital’s electronic medical records for the period spanning January 1, 2023, to December 31, 2023. A total of 89 cases were subjected to statistical evaluation. Frequencies and percentages were determined, while sensitivity, specificity, PPV, and NPV were calculated for TIRADS categories 3, 4, and 5 using FNAC results as the comparative standard. Histopathological findings were also reviewed and analyzed to support the conclusions.

Inclusion criteria

The study population comprised all patients who visited the hospital’s surgical outpatient clinic within the 12-month study period of 2023 and underwent thyroid US. Eligible participants included those referred to US either for non-specific neck symptom evaluation or due to clinically evident thyroid enlargement. Only cases with thyroid nodules detected on US and classified using the TIRADS system were included. Patients with a prior hemithyroidectomy who developed nodules in the remaining thyroid lobe were also considered for inclusion.

Exclusion criteria

The study excluded patients diagnosed with diffuse hyperplastic goiter, those who had undergone total thyroidectomy and were attending follow-up visits, and individuals with thyroid nodules who passed away during the audit period.

## Results

**Figure 1:**
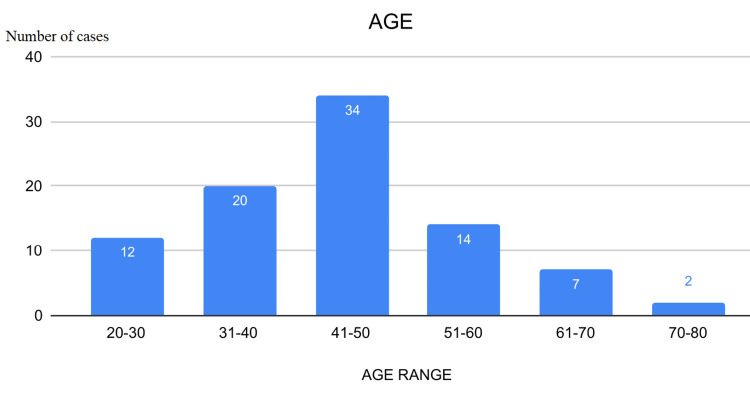
Chart showing age range of cases

**Figure 2:**
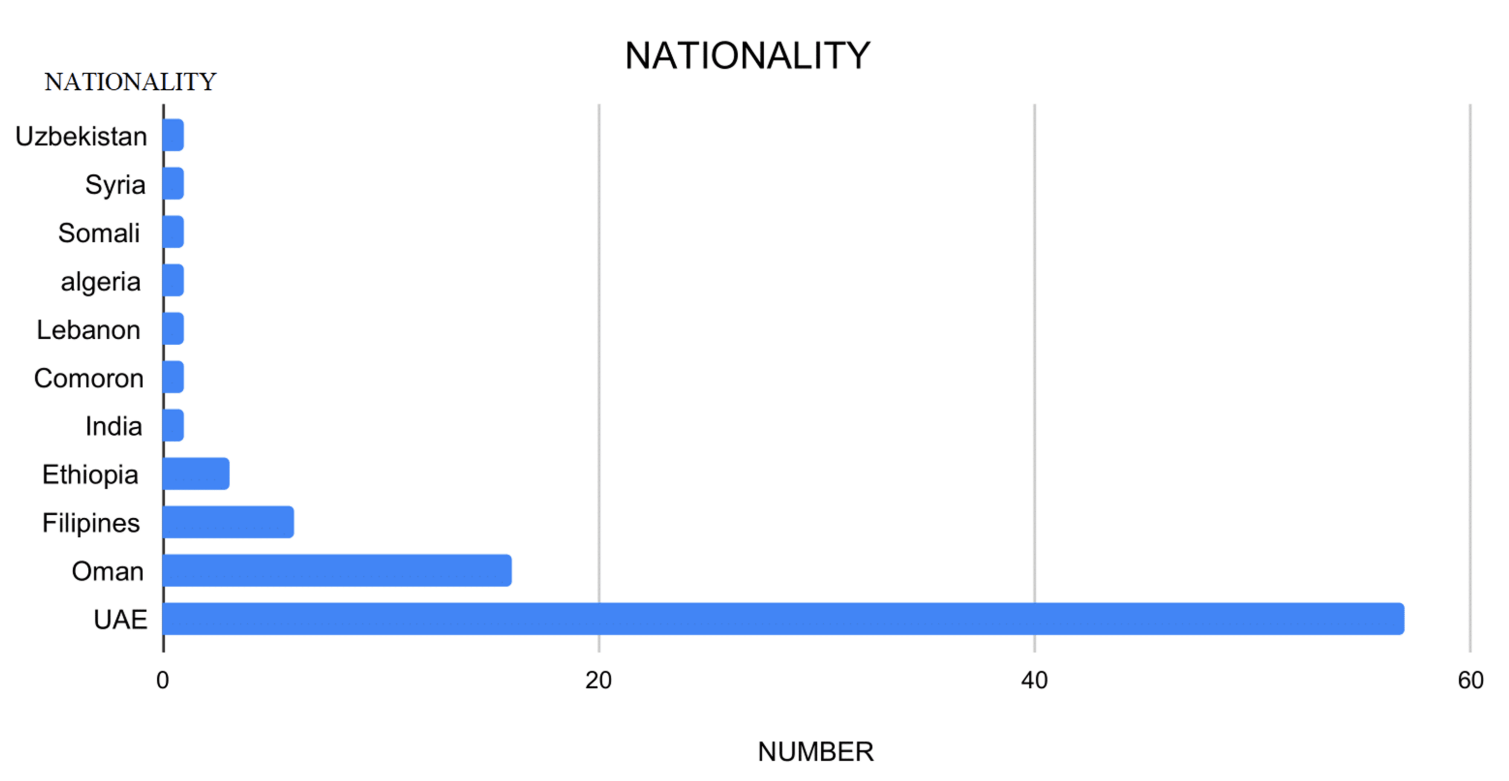
Chart displaying the nationality distribution

**Figure 3:**
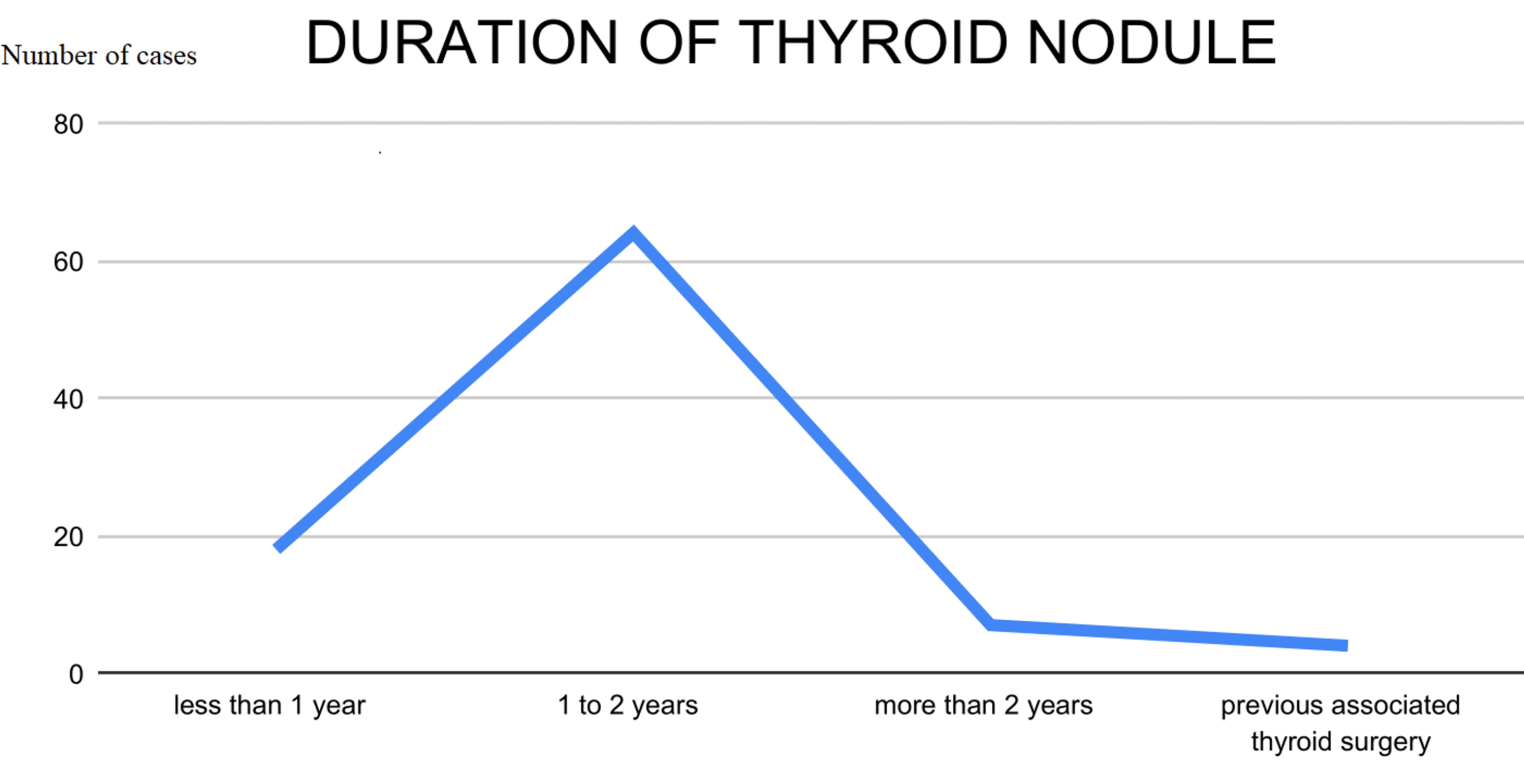
Chart showing duration of thyroid nodules

**Figure 4:**
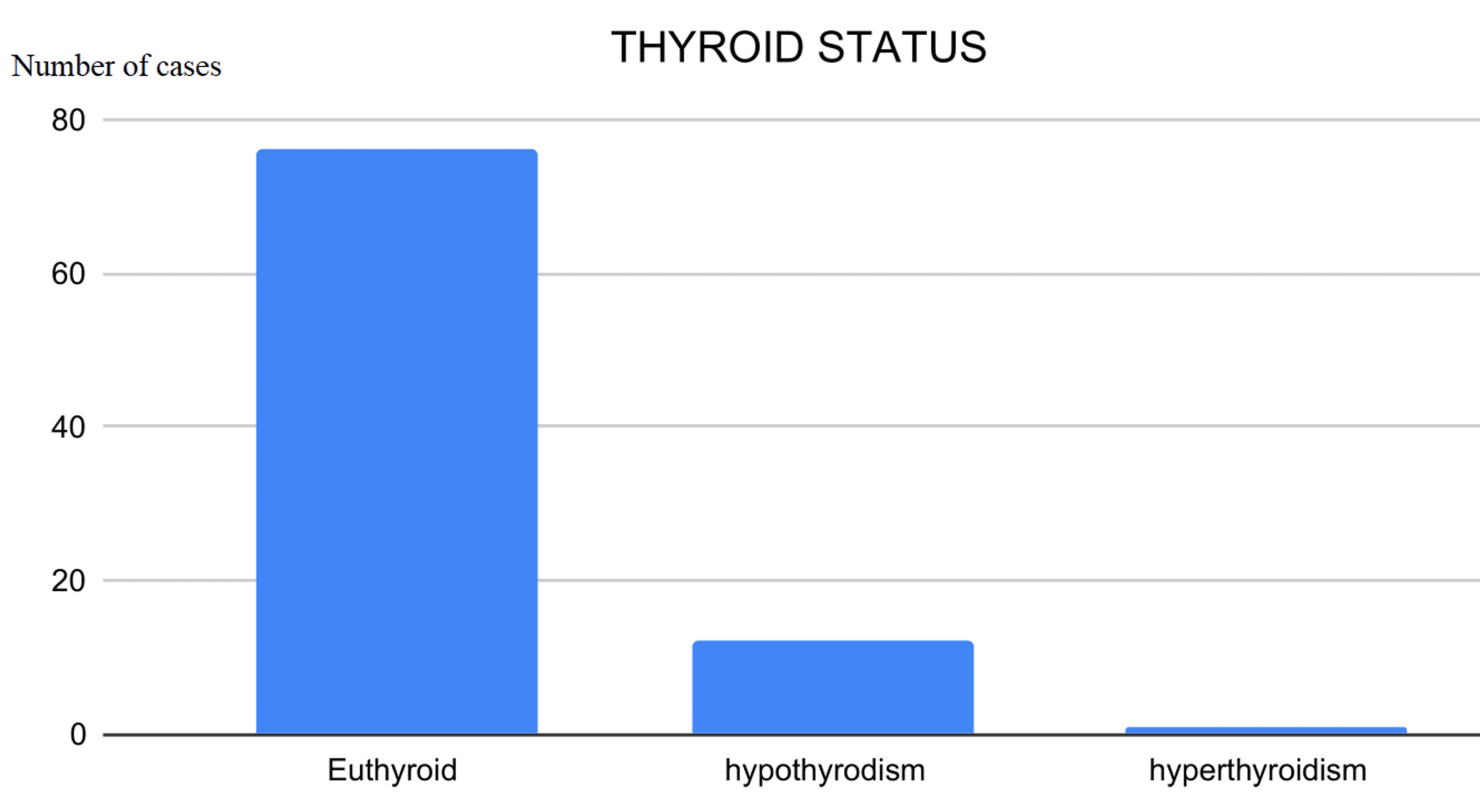
Chart displaying thyroid function status

This observational study analyzed data from 89 patients, examining variables such as age, gender, nationality, symptom duration, thyroid function, US TIRADS score, Bethesda FNAC grade, surgical interventions, and post-surgical histopathology outcomes. Descriptive and biostatistical methods were employed for data analysis. The majority of patients were aged 41-50 years, with the youngest being 20 and the oldest 76 years (Figure 1). UAE nationals constituted 64% of the cohort, followed by Omanis (Figure 2), and 87% of the patients were female. Approximately 72% of patients reported a history of thyroid nodules lasting 1-2 years, 20% had nodules for less than a year, and 8% had nodules for over two years (Figure 3). Four patients had previously undergone thyroid lobectomy. Biochemically, 86% were euthyroid, 13% were hypothyroid, and one patient had hyperthyroidism (Figure 4).

Of the 89 cases, 37 (41.5%) underwent US-guided FNAC (Table 1). In 38 cases (42.5%), clinicians did not recommend FNAC, while in the remaining cases, patients refused the procedure (refusal rate: 27.5%). No FNACs were performed for TIRADS 1 cases. For TIRADS 2, two out of 19 cases (10%) underwent FNAC, with Bethesda grades not exceeding 2. Among TIRADS 3 cases, 22 out of 48 (45%) had FNAC, with 60% reporting Bethesda grade 3 or lower, and no surgical interventions were performed based on FNAC results (Table 1).

**Table 1:** Cumulative data of TIRADS, FNAC Bethesda score, and histopathology TIRADS, Thyroid Imaging Reporting and Data System; FNAC, fine-needle aspiration cytology

TIRADS	Number	FNAC done	FNAC refused	FNAC not recommended	Bethesda grade 1	Bethesda grade 2	Bethesda grade 3	Bethesda grade 4	Bethesda grade 5	Bethesda grade 6	Surgery	Type of surgery	Histopathology
1	3	0	0	3	0	0	0	0	0	0	0	0	0
2	19	2	0	17	1	1	0	0	0	0	0	0	0
3	48	22	8	19	8	13	1	0	0	0	0	0	0
4	18	12	6	0	2	5	1	4	0	0	4	3 - lobectomy, 1 - total thyroidectomy	1 - papillary carcinoma, 1 - follicular adenoma, 2 - colloid goiter
5	1	1	0	0	0	0	0	0	1	0	1	1 - total thyroidectomy	Papillary carcinoma
Total		36	14	39	11	19	2	4	1	0	5		
Grand total	89												

For TIRADS 4, all cases were advised FNAC, but only 12 out of 18 (66%) underwent the procedure, with six refusing. Of these, four (33%) were graded Bethesda 4, while eight were graded Bethesda 3 or lower. All Bethesda 4 cases underwent thyroid lobectomy, with histopathology revealing one papillary carcinoma, one follicular adenoma, and two colloid goiters. The malignancy confirmation rate for TIRADS 4 was 5.5% (one out of 18), and for Bethesda 4, it was 25% (one out of four).

One case (1.12%) was classified as TIRADS 5 (Table 2) and underwent FNAC, which returned as Bethesda grade 5. The patient underwent total thyroidectomy and cervical lymph node dissection, with histopathology confirming papillary carcinoma, demonstrating a 100% diagnostic accuracy for TIRADS 5 combined with Bethesda grade 5.

**Table 2:** Malignancy risk comparison with TIRADS score and Bethesda grading TIRADS, Thyroid Imaging Reporting and Data System; FNAC, fine-needle aspiration cytology

TIRADS score	Bethesda FNAC grade	Surgery percentage	Malignant histopathology	Cancer yield rate with TIRADS score	Cancer yield rate with Bethesda grade 4 and above
1	Not performed	0	Not applicable	Not applicable	Not applicable
2	1 to 2	0	Not applicable	Not applicable	Not applicable
3	Up to 3	0	0	0	0
4	4	100	25%	5.5%	25%
5	5	100	100%	100%	100%

Follow-up schedules varied, with 81% of patients monitored every six months, and the remainder at three-month or one-year intervals. Statistical analysis of TIRADS 3, 4, and 5 as predictive tools for malignancy, compared with FNAC and histopathology, revealed the following: TIRADS 3 exhibited 100% specificity in identifying benign cases. For TIRADS 4, sensitivity was 100% (95% CI: 0.0-1.0), specificity was 82.35% (95% CI: 0.59-0.94), PPV was 25% (95% CI: 0.04-0.69), and NPV was 100% (95% CI: 0.80-1.0). For TIRADS 5, sensitivity and PPV were both 100% (95% CI: 0.0-1.0), though specificity and NPV could not be calculated due to limited data.

TIRADS 4 and 5 demonstrated potential in predicting malignancy, though FNAC results should be interpreted cautiously due to false positives. Combining TIRADS assessment with FNAC findings may enhance diagnostic accuracy, particularly for TIRADS 4 nodules.

## Discussion

This study explored the diagnostic value of the TIRADS classification system in guiding FNAC decisions for thyroid nodules. The majority of the cohort consisted of middle-aged female patients, most with a history of thyroid nodules of one to two years’ duration, and thyroid function was predominantly normal. TIRADS 3 nodules had low clinical significance, as they did not result in surgical intervention, whereas TIRADS 4 and 5 categories showed stronger correlations with suspicious cytology and histopathologically confirmed malignancies. These findings underscore the potential utility of combining TIRADS scoring with FNAC to improve risk stratification and clinical decision-making in evaluating thyroid nodules. A noteworthy observation was that a substantial proportion of patients were reluctant to undergo FNAC.

Malignancy risks associated with TIRADS scoring are well documented in the literature (Table 3) [8].

**Table 3:** Malignancy risks associated with TIRADS scoring Data derived from 327 operated thyroid cases.

TIRADS score	Malignancy risk, %
1&2	Nil
3	<5%
4	5%-85% (average 30%)
5	85%-100%

A study by Negro R, Greco G, et al. [9] demonstrated that ultrasound-based risk stratification, particularly for nodules with TIRADS scores of 3 and above, significantly enhances malignancy detection, independent of nodule size. This underscores the value of TIRADS scoring in guiding clinical decisions. However, addressing patient concerns about the potential complications of FNAC remains a major challenge for healthcare providers. Several studies [4,8-10] advise against routine FNAC without prior risk assessment using TIRADS scoring to minimize unnecessary procedures.

Patients with TIRADS 1 or 2 nodules should avoid FNAC, as their malignancy risk is negligible; instead, follow-up ultrasounds at six to 12 months are recommended [6], aligning with our findings. Notably, 60% of FNAC procedures in our study were performed on patients with TIRADS 3 scores, none of whom required surgery, suggesting that FNAC may be unnecessary for this group. While some studies advocate immediate surgery for Bethesda grade 3 cases, we opted for observation and follow-up ultrasounds. Decision-making for TIRADS 3 cases, with a malignancy risk below 5%, could be refined using additional factors such as nodule size (>20 mm), interval growth, advanced age, and male sex [7,10].

For TIRADS 4 and 5 nodules, ultrasound-guided FNAC is recommended [4,11]. However, for patients declining FNAC, close monitoring every three to six months is advised [4,6-12]. In our study, TIRADS 4 cases with Bethesda grade 4 had a malignancy risk of 25%, while a TIRADS 5 case with Bethesda grade 5 was confirmed as papillary carcinoma, demonstrating 100% specificity [7,9,12-14]. The 2023 European Thyroid Association Clinical Practice Guidelines suggest FNAC based on nodule size cutoffs: 20 mm for TIRADS 3, 15 mm for TIRADS 4, and 10 mm for TIRADS 5 [4,14,15].

In our study, nodule size was not consistently reported, limiting its role in decision-making. Despite this limitation, our findings align with those of George NA et al. [16]. For TIRADS 5 nodules, regardless of FNAC results, and for Bethesda 4 and 5 cases, regardless of TIRADS score, surgical intervention should be strongly considered due to the high malignancy risk [17].

## Conclusions

This study concludes that performing routine FNAC for TIRADS categories 1 and 2 is unnecessary, as these nodules carry an extremely low risk of malignancy. Similarly, FNAC was found to be generally unwarranted for TIRADS 3 nodules in our cohort, as none of the patients in this category required surgical management. However, selective use of FNAC may be appropriate for TIRADS 3 nodules in specific cases, especially when clinical factors such as patient age or nodule size raise concern. In contrast, FNAC was routinely performed for TIRADS 4 nodules in this study and deemed crucial for TIRADS 5 nodules due to their high likelihood of malignancy. The study also observed that a notable proportion of patients refused FNAC, primarily due to anxiety about pain or procedural complications. A key limitation was the unavailability of data on nodule size, which limited the study’s ability to assess certain decision-making parameters. Nevertheless, the results are consistent with prior research, supporting the role of the TIRADS classification system in guiding FNAC decisions. Based on our findings, we recommend periodic follow-up with thyroid US at six-month intervals for continued monitoring of thyroid nodules.
